# Condensate-based sequestration enables high-efficiency synthesis of mussel foot protein Mcofp-3

**DOI:** 10.1128/aem.00786-26

**Published:** 2026-06-04

**Authors:** Litao Hu, Liyan Chen, Yan Wang, Siting Yu, Sen Xiao, Wuxia Chen, Jianhua Cheng, Zhen Kang

**Affiliations:** 1 College of Environment and Energy, South China University of Technology 26467, Guangzhou, China; 2 Institute of Future Food Technology, JITRI 718199 , Yixing, China; 3 The Key Laboratory of Carbohydrate Chemistry and Biotechnology, Ministry of Education, School of Biotechnology, Jiangnan University, Wuxi, China; 4 The Science Center for Future Foods, Jiangnan University 66374, Wuxi, China; Danmarks Tekniske Universitet, Kgs. Lyngby, Denmark

**Keywords:** cytotoxicity alleviation, hyaluronic acid, condensate-based sequestration, mussel foot proteins

## Abstract

Mussel foot proteins (Mfps) hold immense potential for medicine and bioengineering, owing to their exceptional adhesion and biocompatibility. However, traditional extraction from mussels is low-yield and ecologically unsustainable. While microbial synthesis offers a green alternative, the intrinsic hydrophobicity and adhesiveness of Mfps often induce severe host cytotoxicity, resulting in low yields. Initially, attempts to express Mcofp-3 in *Escherichia coli* were limited to 60 mg L^−1^ due to such cytotoxic effects. To overcome this bottleneck, we developed a hyaluronic acid (HA)-driven “condensate-based sequestration” strategy. This approach leverages electrostatic interactions to sequester Mcofp-3 into condensates, thereby shielding the host cells and alleviating metabolic stress. This strategy significantly restored cell viability, increasing the titer to 460 mg L^−1^ in shake flasks. Moreover, the process was successfully scaled up to a 50 L fed-batch fermentation, achieving a final titer of 1.6 g L^−1^. Following production, *in vitro* 3,4-dihydroxyphenylalanine (DOPA) modification was performed using immobilized TyrVs-CipA, yielding functional Mcofp-3 that exhibited anti-inflammatory activity and enhanced cell migration capabilities. This work establishes a versatile, green paradigm for the high-yield production of proteins with low yields and increased toxicity in bacterial expression systems.

**IMPORTANCE:**

Mussel foot proteins (Mfps) are prized bio-adhesives for medical applications, yet their supply remains limited. Harvesting mussels is environmentally unsustainable, while microbial synthesis is challenging due to the severe toxicity of these proteins to host cells. In this study, we developed a “condensate-based sequestration” strategy to overcome this bottleneck. By employing hyaluronic acid to isolate the toxic proteins within the bacteria, we shielded the host cells and achieved high-yield production. This approach enabled the gram-scale synthesis of modified Mfps with desirable bioactivity. Beyond synthesizing Mfps, this strategy establishes a versatile, green platform for manufacturing a wide range of other “difficult-to-express” toxic proteins that are currently commercially unviable.

## INTRODUCTION

Mussel foot proteins (Mfps) are natural adhesive proteins secreted by marine mussels, enabling robust underwater adhesion to diverse substrates under harsh marine conditions, including high salinity and water turbulence (1–4). Their outstanding adhesion relies on synergistic non-covalent interactions and ordered self-assembly, forming stable, biocompatible, and biodegradable interfacial structures (5–7). As such, Mfps are ideal candidates for biomedical adhesives, wound repair materials, tissue engineering scaffolds, and eco-friendly antifouling coatings (8–11). However, current Mfps production mainly depends on direct extraction from mussels, which is unsustainable and resource-intensive. Mfps are present at extremely low levels in mussel tissues, requiring hundreds of kilograms of raw mussels to obtain merely several grams of target protein, leading to high costs and severe marine ecological pressure (12). Therefore, developing alternative, resource-independent production technologies is urgently needed in this field.

Recombinant expression in microbial systems represents a feasible green route for the efficient and sustainable production of Mfps, enabling controllable and large-scale biosynthesis using renewable carbon sources (13–17). To date, Mfps have been successfully heterologously expressed in multiple microbial hosts, including *Escherichia coli* (10, 14, 18–20), *Bacillus subtilis* (21), and *Pichia pastoris* (22). For instance, Cha et al. (23) realized the recombinant expression of Mgfp-5 from *Mytilus galloprovincialis* in *E. coli* via codon optimization, with a yield of 50 mg L^−1^. Another study achieved efficient secretory expression of Mfp3 and Mfp5 in *B. subtilis* through a SpyTag-mediated fusion strategy, reaching titers of 255 mg L^−1^ and 119 mg L^−1^, respectively (21). These studies confirm that strategies, including sequence optimization, fusion tag design, and secretory expression, can enhance Mfps production to a certain extent.

However, current production levels are still far from meeting industrial demand. The core bottleneck is the intrinsic properties of Mfps: high hydrophobicity, abundant repetitive sequences, and large numbers of cationic amino acids, which easily trigger severe cytotoxicity, insoluble inclusion body formation, and distinct host growth inhibition in conventional hosts, such as *E. coli* (14, 24, 25). To improve expression, researchers commonly adopt fusion expression strategies, such as constructing fp-151 by fusing fp-1 repeats at both ends of fp-5, or designing multi-domain tandem proteins like fp-535 (26, 27). Although these approaches boost expression yields, they inevitably alter the native protein sequence and impair its intrinsic biological function (21, 28, 29). Therefore, efficiently alleviating intracellular toxicity and achieving high-level production while preserving the complete native sequence of Mfps remains a critical scientific challenge in this field.

To overcome this bottleneck, we developed a hyaluronic acid (HA)-mediated “condensate-based sequestration” strategy. We engineered the host to synthesize HA within the cytoplasm, where HA subsequently underwent liquid-liquid phase separation (LLPS) to form a condensed phase. These compartments electrostatically recruit and sequester soluble *Mytilus coruscus* foot protein 3 (Mcofp-3), effectively shielding the host cell and alleviating toxicity. This approach boosted production to 460 mg L^−1^ in shake flasks and achieved a titer of 1.6 g L^−1^ in a 50 L fed-batch fermentation. Furthermore, we constructed an immobilized tyrosinase, TyrVs–CipA, leveraging the self-assembling CipA tag to enable efficient *in vitro* 3,4-dihydroxyphenylalanine (DOPA) modification. The resulting modified protein exhibited anti-inflammatory activity and significantly enhanced keratinocyte migration, underscoring its biomedical potential. Collectively, this work presents a sustainable strategy for the biosynthesis of cytotoxic adhesive proteins and offers a versatile framework for the expression of other challenging recombinant proteins.

## RESULTS

### Expression and purification of Mcofp-3 in recombinant *E. coli*

**Fig 1:**
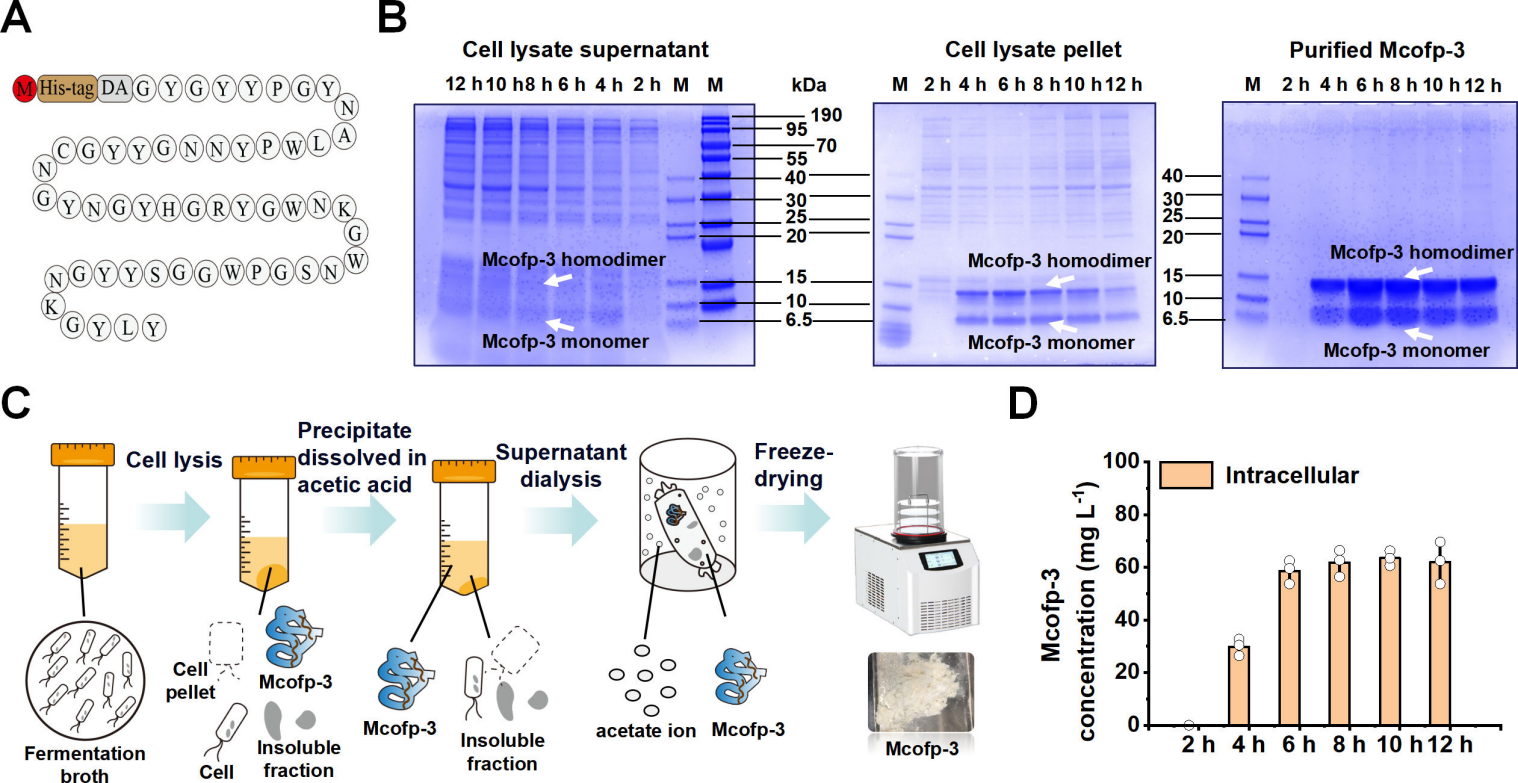
Recombinant expression and purification of Mcofp-3 in *E. coli*. (**A**) Schematic representation of the Mcofp-3 amino acid sequence. (**B**) SDS-PAGE analysis of cell lysates and purified protein from the recombinant strain EC-Mcofp-3: 1, supernatant; 2, pellet; 3, purified Mcofp-3. (**C**) Purification: after fermentation, cells were harvested by centrifugation and lysed; the insoluble pellet was solubilized in 25% acetic acid, dialyzed against water using a 3 kDa cutoff membrane to remove salts and small molecules, and finally lyophilized to obtain purified Mcofp-3. (**D**) Intracellular yield of Mcofp-3 in recombinant strain EC-Mcofp-3. Data are presented as mean ± SD (*n* = 3 independent replicates), and the results shown are representative of three independent experiments with similar results.

To improve expression efficiency, the gene encoding foot protein-3 from the thick-shell mussel (*M. coruscus*), designated Mcofp-3, was codon-optimized (Fig. 1A) and cloned into the expression vector pET-32a (+). The resulting construct was transformed into *E. coli* BL21(DE3), yielding the recombinant strain EC-Mcofp-3. Following shake-flask fermentation, cells were harvested and separated into supernatant and pellet fractions. SDS-PAGE analysis revealed that Mcofp-3 was predominantly expressed as inclusion bodies in the pellet, with only a small fraction present in the soluble supernatant (Fig. 1B). Distinct protein bands were observed at approximately 7.5 and 15 kDa, corresponding to the theoretical molecular weights (MWs) of the Mcofp-3 monomer and dimer, respectively, confirming successful expression of the target protein (Fig. 1B).

Leveraging the known solubility of mussel foot proteins in acetic acid (14, 20), we established an efficient purification protocol (Fig. 1C; Fig. S1). SDS-PAGE confirmed that this method yielded highly pure Mcofp-3 (Fig. 1B). Furthermore, time-course fermentation analysis showed that Mcofp-3 production plateaued between 6 and 12 h, reaching a final titer of approximately 60 mg L^−1^ (Fig. 1D).

### Impact of Mcofp-3 expression on growth and metabolic activity in recombinant *E. coli*

**Fig 2:**
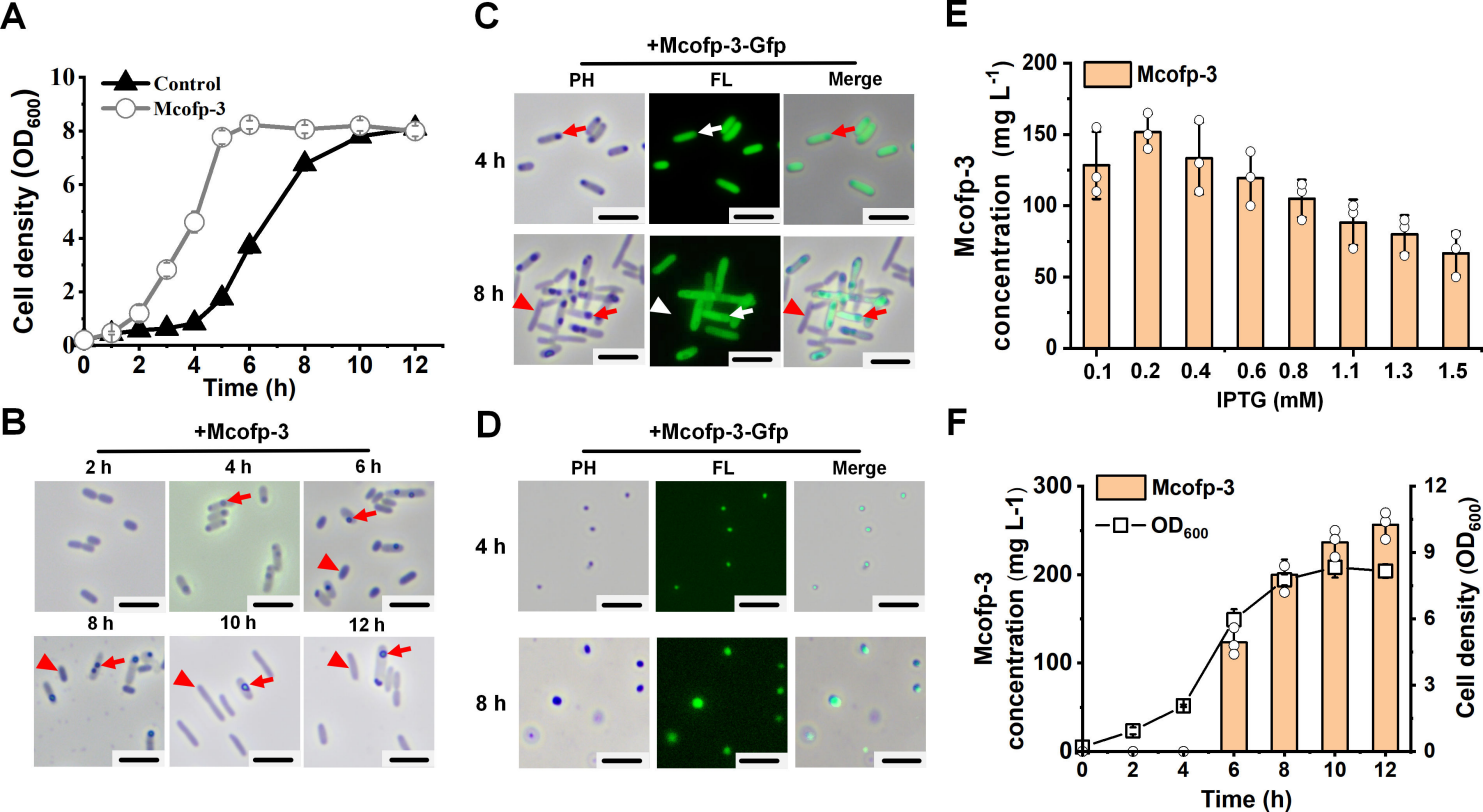
Impact of Mcofp-3 expression on *E. coli* metabolism and process optimization. (**A**) Growth curves of strains expressing Mcofp-3, with strains harboring an empty vector serving as the negative control. (**B**) Phase-contrast microscopy images of recombinant strain EC-Mcofp-3 at different growth stages; arrows indicate intracellular mussel protein aggregates, while arrowheads denote cells lacking Mcofp-3. (**C**) Phase-contrast and fluorescence microscopy images of the strain EC-Mcofp-3-GFP. (**D**) Phase-contrast and fluorescence microscopy images of precipitates obtained from lysed EC-Mcofp-3-GFP cells. (**E**) Mcofp-3 yield following optimization of IPTG concentration (induction performed at 37°C for 1 h). (**F**) Cell density (OD_600_) and Mcofp-3 yield of the strain EC-Mcofp-3 under optimal fermentation conditions determined by systematic optimization (3 h induction, 0.2 mM IPTG, 30°C). Scale bars: 5 μm, all data are presented as mean ± S.D. from three independent biological replicates (*n* = 3). Microscopy experiments were performed in triplicate with representative results shown.

To investigate why Mcofp-3 production plateaued between 6 and 12 h, we monitored cell growth dynamics and morphological changes (Fig. 1D). Results showed that IPTG induction caused an immediate arrest in cell growth, a state that persisted for approximately 3 h, indicating a significant metabolic burden imposed by the heterologous protein expression (Fig. 2A). Phase-contrast microscopy revealed the appearance of intracellular high-refractive granules; these granules were present in nearly all cells within the first 4 h (>90% aggregate-positive cells) but gradually declined in number over time <15% by 12 h (Fig. 2B; Fig. S2).

To confirm the identity of these granules, we constructed an Mcofp-3–GFP fusion expression system. Fluorescence microscopy demonstrated that the green fluorescent signal was observed in these granules. Furthermore, strong fluorescence was retained in the pellet fraction after cell lysis and centrifugation, verifying that the granules were indeed Mcofp-3 aggregates (Fig. 2C and D; Fig. S3). Additionally, soluble Mcofp-3 was detected, uniformly distributed throughout the cytoplasm, consistent with the aforementioned SDS-PAGE results (Fig. 1B and 2C). Notably, fluorescence tracking further revealed the spatiotemporal heterogeneity of expression: during the early induction phase, almost all cells expressed Mcofp-3 (evidenced by strong fluorescence); however, in the late fermentation stage, the fluorescent signal disappeared in the majority of cells, indicating that protein synthesis had ceased (Fig. S3). In summary, Mcofp-3 is synthesized efficiently during the early fermentation stage—primarily as aggregates with a minor soluble fraction—but synthesis ceases completely in later stages, directly causing the production plateau (Fig. 1D and 2B).

**Fig 3:**
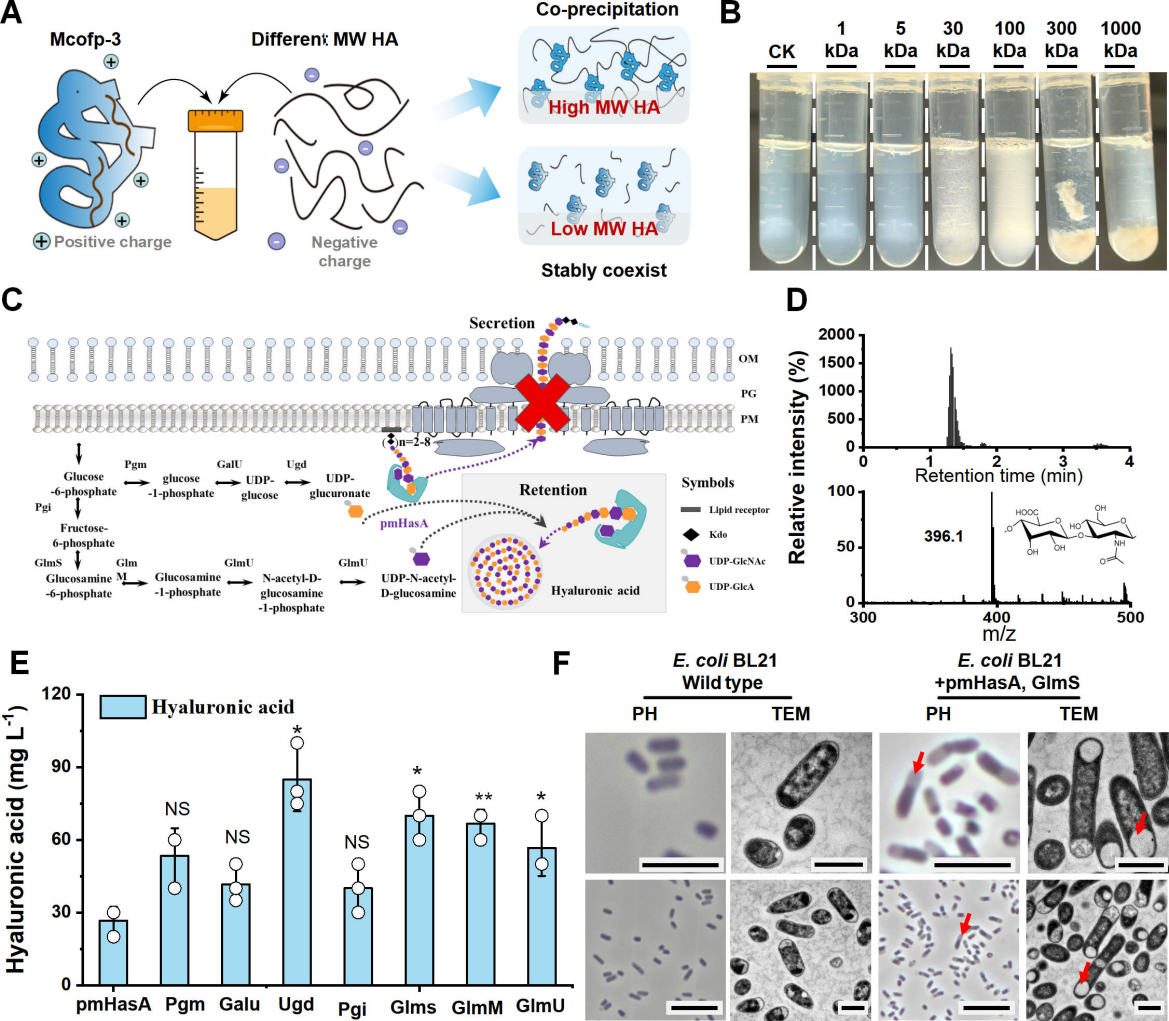
Interactions between HA of varying MWs and Mcofp-3, and the construction of an intracellular HA synthesis pathway. (**A**) Schematic illustration showing that high-MW HA forms precipitates upon interaction with Mcofp-3, whereas low-MW HA remains soluble and coexists with the protein. (**B**) Visual confirmation of these interactions; mixtures containing 5 g L^−1^ HA and 1 g L^−1^ Mcofp-3 display distinct phase behaviors depending on the HA molecular weight. (**C**) Schematic of the HA biosynthesis and transport model, comprising three stages: initiation (glycolipid primer formation), polymerization (sequential chain extension), and secretion (ABC transporter-mediated export). Notably, in the absence of a functional transport system, HA accumulates intracellularly. pmHasA, hyaluronan synthase; Pgm, phosphoglucomutase; GalU, glucose-1-phosphate uridylyltransferase; Ugd, UDP-glucose 6-dehydrogenase; Pgi, glucose-6-phosphate isomerase; GlmS, L-glutamine-D-fructose-6-phosphate aminotransferase; GlmM, phosphoglucosamine mutase; GlmU, UDP-N-acetylglucosamine pyrophosphorylase/glucosamine-1-phosphate N-acetyltransferase. (**D**) Verification of the chemical identity of synthesized HA by liquid chromatography-mass spectrometry (LC-MS), showing extracted ion chromatograms and mass spectra. (**E**) Detection of HA in the cell pellets of engineered *E. coli* strains featuring enhanced UDP-GlcA and UDP-GlcNAc pathways. Statistical evaluation (*P* value) compared to strain EC01 (pmHasA) was performed by a two-sided *t*-test. *P < 0.05, **P < 0.01, ***P < 0.001; NS, not significant (*P* ≥ 0.05). (**F**) Phase-contrast and transmission electron microscopy (TEM) micrographs comparing wild-type *E. coli* with cells expressing *pmHasA* and *glmS*, revealing morphological differences. Scale bars: 5 μm (PH) and 1 μm (TEM). All data represent the mean ± S.D. from three independent biological replicates (*n* = 3).

To alleviate this metabolic burden and enhance production, we systematically optimized induction time, IPTG concentration, and post-induction temperature. Induction at 3 h yielded the highest titer (130 mg L^−1^), outperforming earlier (1 h) or later induction (Fig. S4). Reducing IPTG concentration from the standard 1 to 0.2 mM and lowering the cultivation temperature from 37°C to 30°C further improved expression (Fig. 2E; Fig. S4), likely by moderating translation rates and avoiding premature protein overload. Under the optimal conditions (3 h induction, 0.2 mM IPTG, 30°C), Mcofp-3 production reached 250 mg L^−1^ (Fig. 2F). Nevertheless, a substantial fraction of cells still showed no detectable Mcofp-3 expression in late fermentation stages (Fig. S5), indicating that conventional process optimization alone cannot fully overcome the inherent cytotoxicity of Mcofp-3. Thus, the protein’s intrinsic interference with host physiology remains the fundamental bottleneck limiting high-level production.

### Hyaluronic acid and Mcofp-3 interact to form insoluble complexes

Given that the cytotoxicity of Mcofp-3 is the main barrier to high-yield production, we hypothesized that co-expressing a macromolecule that specifically binds Mcofp-3 could promote its aggregation and reduce free protein levels, thereby alleviating cytotoxicity. Given the abundance of cationic residues on the surface of Mcofp-3, we screened a panel of multivalent polyanionic polymers—including HA, chondroitin sulfate, heparin, and sodium alginate—to evaluate their ability to engage in electrostatic and non-covalent interactions with Mcofp-3. Our results demonstrated that Mcofp-3 interacted with HA, chondroitin sulfate, heparin, and sodium alginate to form visible precipitates, whereas no obvious precipitation was detected upon incubation with dextran or polyphosphoric acid (Fig. 3A and B; Fig. S6). Further investigation using HA of varying MWs revealed a clear binding preference of Mcofp-3 for high-MW species: distinct flocculent precipitates formed with 1,000 and 300 kDa HA, fine precipitates with 100 and 30 kDa HA, and no precipitation was observed with 5 and 1 kDa HA (Fig. 3A and B). This difference likely stems from the multivalent polyanionic nature and flexible chain conformation of high-MW HA (30, 31), which enable multipoint electrostatic and non-covalent interactions with the cationic residues (e.g., lysine, arginine) abundant in Mcofp-3, leading to networked crosslinking and phase separation (Fig. 3A). In contrast, low-MW HA lacks sufficient valency and chain length to support effective multivalent binding, resulting only in soluble complexes.

To test whether HA can bind Mcofp-3 intracellularly and reduce its free concentration—thereby mitigating metabolic stress—we first reconstructed the HA biosynthetic pathway in *E. coli* BL21(DE3). Noting that BL21 lacks a functional ABC transporter system for polysaccharide export (32, 33), we employed the type II hyaluronan synthase from *Pasteurella multocida* (pmHAS), which catalyzes HA chain elongation directly at the cytoplasmic face of the inner membrane, enabling intracellular HA synthesis and accumulation (Fig. 3C). Liquid chromatography-mass spectrometry (LC-MS) analysis confirmed HA production in the cytoplasm, albeit at low initial levels (Fig. 3D).

To enhance HA biosynthesis (34, 35), we overexpressed the genes responsible for synthesizing the nucleotide sugar precursors UDP-glucuronic acid (UDP-GlcA) and UDP-N-acetylglucosamine (UDP-GlcNAc). This included the upregulation of *pgm*, *galU*, and *ugd* to boost UDP-GlcA levels and *pgi*, *glmS*, *glmM*, and *glmU* to enhance UDP-GlcNAc availability (Fig. 3D). Overexpression of *ugd* (UDP-glucose dehydrogenase), in particular, significantly boosted HA yield (Fig. 3E). Notably, the synthesized HA spontaneously underwent LLPS in the cytoplasm, forming dense, hydrophilic condensates—consistent with its high-MW and polyelectrolyte character (Fig. 3F). However, excessive HA accumulation exerted physical pressure on the cell envelope, often causing lysis due to the limited mechanical strength of the *E. coli* cell envelope (33, 36) (Fig. S7). Therefore, we selected strain EC06 (pmHasA + GlmS), which produces HA with a MW of 410 kDa (Fig. S8) and maintains good cell integrity, as the chassis for subsequent Mcofp-3 co-expression.

### Co-engineering HA biosynthesis mitigates Mcofp-3 toxicity and enhances its production

**Fig 4:**
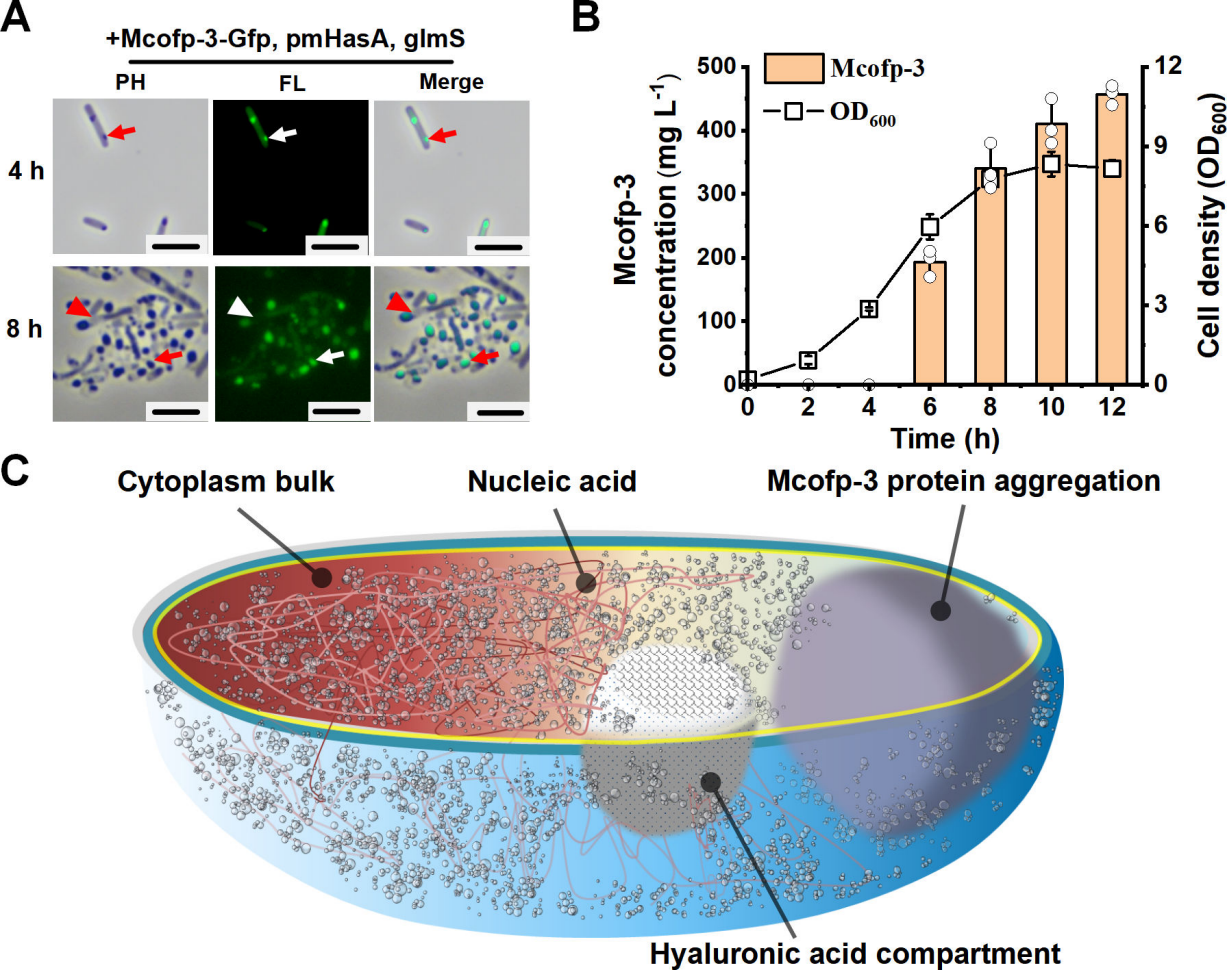
The “condensate-based sequestration” strategy promotes Mcofp-3 synthesis. (**A**) Phase-contrast and fluorescence microscopy images of the engineered strain EC10 co-expressing *mcofp-3-GFP*, *pmHasA*, and *glmS*, scale bars: 5 μm. (**B**) Time-course profiles of OD_600_ and Mcofp-3 yield for the strain co-expressing *mcofp-3*, *pmHasA*, and *glmS*. (**C**) Schematic illustration of the “condensate-based sequestration” strategy. In this model, HA undergoes phase separation to form condensed droplets, which subsequently promote the aggregation of Mcofp-3 into distinct assemblies. All data represent the mean ± S.D. from three independent biological replicates (*n* = 3).

To evaluate the impact of intracellular HA condensates on Mcofp-3 expression, we co-expressed Mcofp-3–GFP (C-terminally fused with GFP) in the engineered *E. coli* strain capable of accumulating HA in the cytoplasm. Fluorescence microscopy revealed that, compared to the control strain EC-Mcofp-3 expressing Mcofp-3 alone, the HA co-expressing strain exhibited a significantly higher proportion of Mcofp-3-positive cells at late fermentation stages (Fig. 4A; Fig. S9). Notably, in the vast majority of cells, GFP fluorescence was concentrated in distinct granular structures rather than diffusely localized in the cytosol (Fig. 4A), indicating that HA condensates effectively sequestered Mcofp-3 into aggregates and reduced its free concentration.

Growth curve analysis further showed that, upon IPTG induction, the co-expression strain did not experience growth arrest and continued to grow steadily (Fig. 4B). Concurrently, Mcofp-3 accumulation increased progressively throughout the mid-to-late fermentation stages without reaching a plateau, strikingly. These results support our hypothesis that the co-expression of HA drives the targeted aggregation of Mcofp-3 and reduces the pool of soluble protein, thereby creating a protective intracellular microenvironment to alleviate metabolic toxicity (Fig. 4C).

### Fed-batch fermentation for the production of Mcofp-3

**Fig 5:**
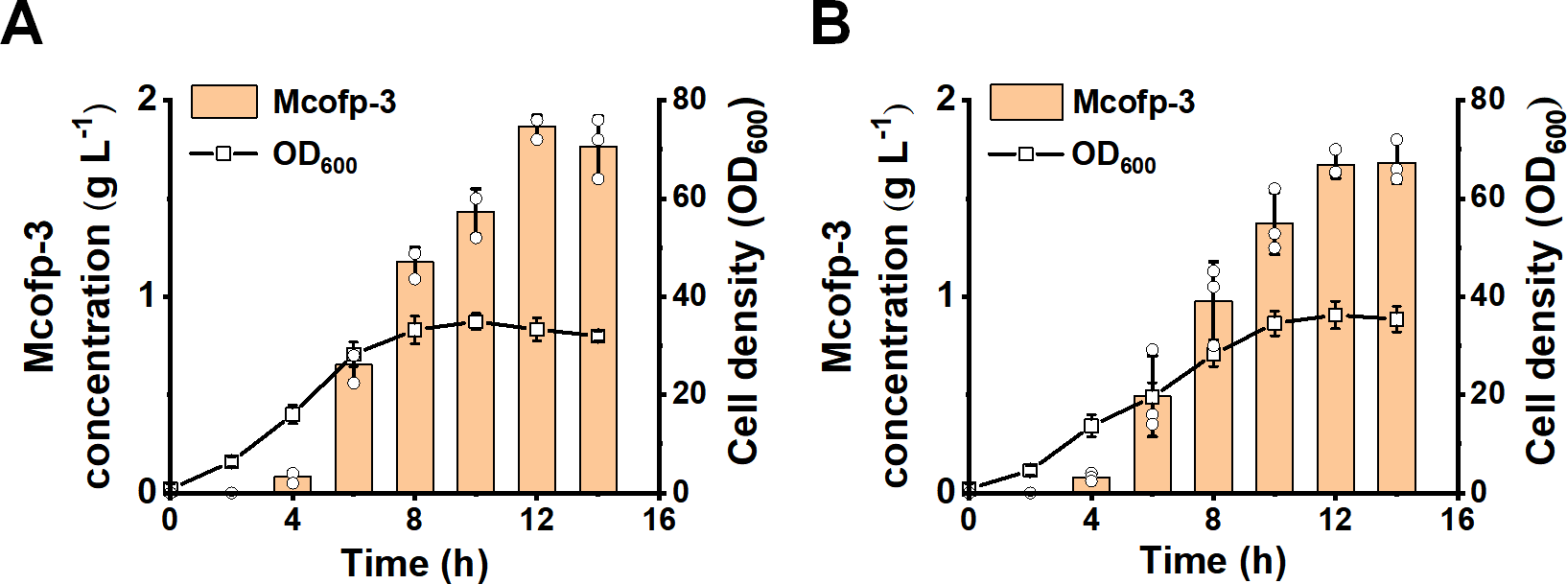
Fed-batch fermentation of Mcofp-3. (**A and B**) Fed-batch cultivation of strain EC09 in 5-L (**A**) and 50-L (**B**) fermenters. The plots show real-time profiles of cell density (OD_600_) and Mcofp-3 yield. All data represent the mean ± S.D. from three independent biological replicates (*n* = 3).

The biosynthetic capacity of the engineered strain EC09 was evaluated in batch fermentation. Cell biomass stabilized between 8 and 14 h, reaching a maximum OD_600_ of 35, while Mcofp-3 continued to accumulate, peaking at 1.7 g L^−1^ at 12 h (Fig. 5A). To further assess scalability, a 50 L bioreactor-scale fermentation was performed. Under these conditions, the maximum OD_600_ reached 40, and Mcofp-3 production peaked at 1.6 g L^−1^ at 14 h (Fig. 5B). These results demonstrate that the HA co-expression system maintains high productivity and process stability upon scale-up, highlighting its strong potential for industrial application. Furthermore, using our established acetic acid-based purification process, we performed pilot-scale recovery from the 50 L fermentation broth. The optimized protocol efficiently removed host proteins, nucleic acids, and other impurities, yielding high-purity Mcofp-3 and enabling future scale-up and applications (Fig. S10).

### Construction of an immobilized tyrosinase system for DOPA modification of Mcofp-3

**Fig 6:**
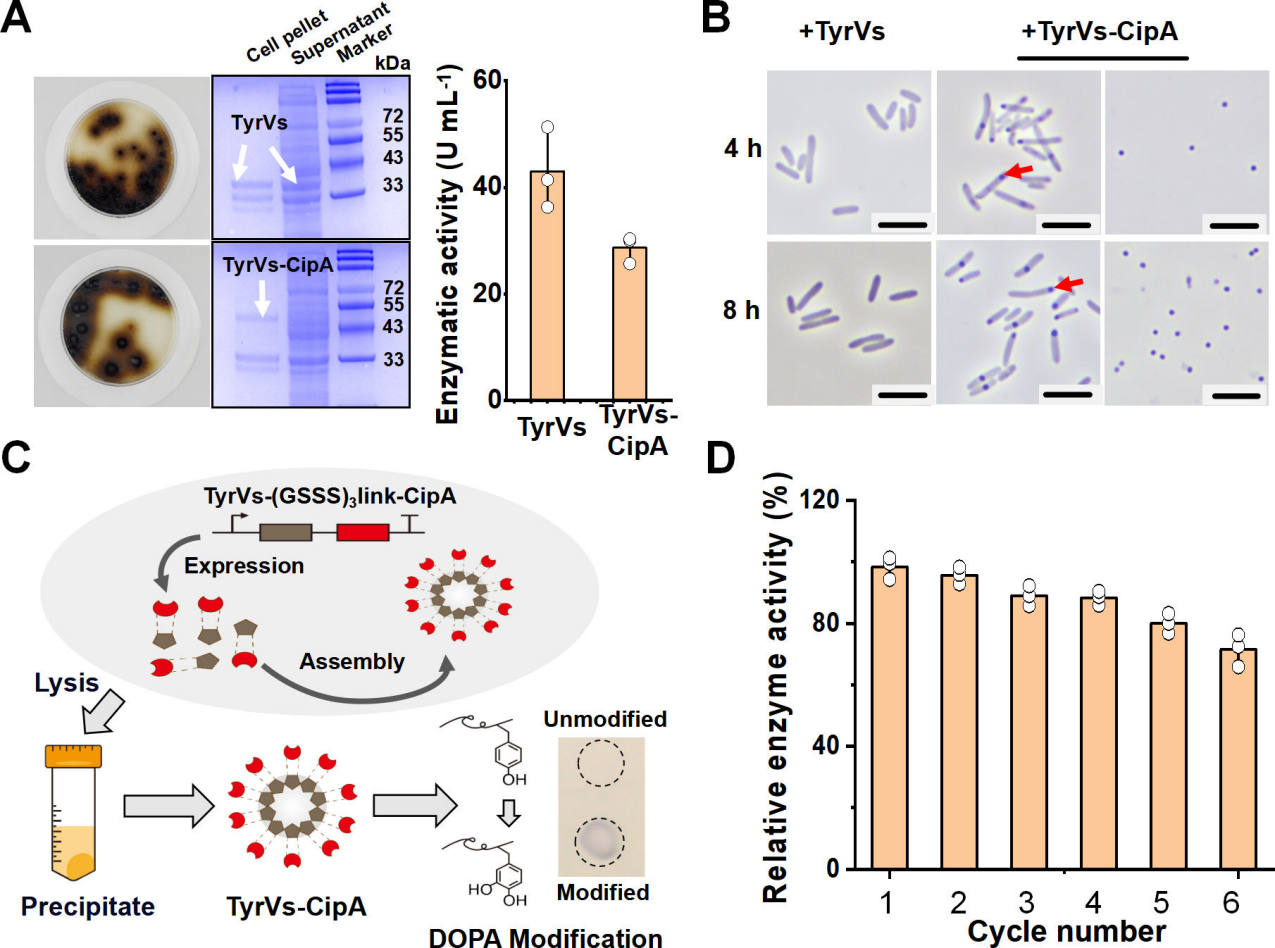
CiPA-based immobilization of tyrosinase and its application in DOPA modification of Mcofp-3. (**A**) Plate-based colorimetric assays of TyrVs and TyrVs-CiPA expressed in *E. coli* BL21(DE3). The tyrosinase catalytic cascade (tyrosine→L-DOPA→dopaquinone→melanin) results in black pigmentation on plates containing active enzyme. Expression levels and catalytic activities were analyzed by SDS-PAGE and activity assays, respectively. (**B**) Phase-contrast microscopy images of *E. coli* expressing TyrVs, TyrVs-CiPA, and the insoluble fraction obtained after cell lysis of TyrVs-CiPA. Scale bars: 5 μm. (**C**) Schematic illustration of TyrVs-CiPA expression, purification, and modification. TyrVs-CiPA spontaneously aggregates intracellularly; the resulting precipitate is collected after cell lysis for DOPA modification. Successful modification of Mcofp-3 is confirmed by a purple color change in the qualitative nitroblue tetrazolium (NBT) assay. (**D**) Reusable DOPA modification of Mcofp-3 using immobilized TyrVs-CiPA. After each modification cycle, the immobilized enzyme was recovered by centrifugation and reused in a fresh Mcofp-3 system. All data are presented as mean ± S.D. from three independent biological replicates (*n* = 3).

Mussel foot proteins are naturally rich in tyrosine residues, which upon hydroxylation yield DOPA—the key functional moiety responsible for their strong wet adhesion, interfacial stability, and diverse biological activities (24). To enable controlled *in vitro* DOPA modification of Mcofp-3, we successfully expressed tyrosinase from *Verrucomicrobium spinosum* (TyrVs) in *E. coli* BL21(DE3). Expression and catalytic activity were confirmed by SDS-PAGE, plate-based colorimetric assays (13) (tyrosine→L-DOPA→dopaquinone→melanin cascade), and spectrophotometric enzyme activity measurements (Fig. 6A).

To enhance enzyme recyclability and operational stability, we constructed a fusion expression vector linking TyrVs with the self-assembling tag protein CipA (Fig. 6A through C). CipA spontaneously forms insoluble intracellular proteinaceous inclusion bodies (37, 38), enabling *in situ* immobilization of the enzyme (Fig. 6B; Fig. S11). Following cell lysis and centrifugation, the pellet was directly used as the immobilized enzyme TyrVs–CipA. Activity assays confirmed that immobilization did not significantly compromise TyrVs functionality (Fig. 6A).

Using this immobilized system, recombinant Mcofp-3 was successfully hydroxylated *in vitro* (Fig. 6C). DOPA modification was verified by nitroblue tetrazolium (NBT) staining: oxidized DOPA (o-quinone) reacts with NBT to form an insoluble purple formazan precipitate. Quantitative colorimetric analysis indicated a DOPA modification degree of approximately 7% (Fig. 6C). Notably, the immobilized enzyme retained >70% of its initial activity after six consecutive batch cycles (Fig. 6D), demonstrating excellent operational stability and reusability.

### Evaluation of the multifunctional bioactivities of Mcofp-3

**Fig 7:**
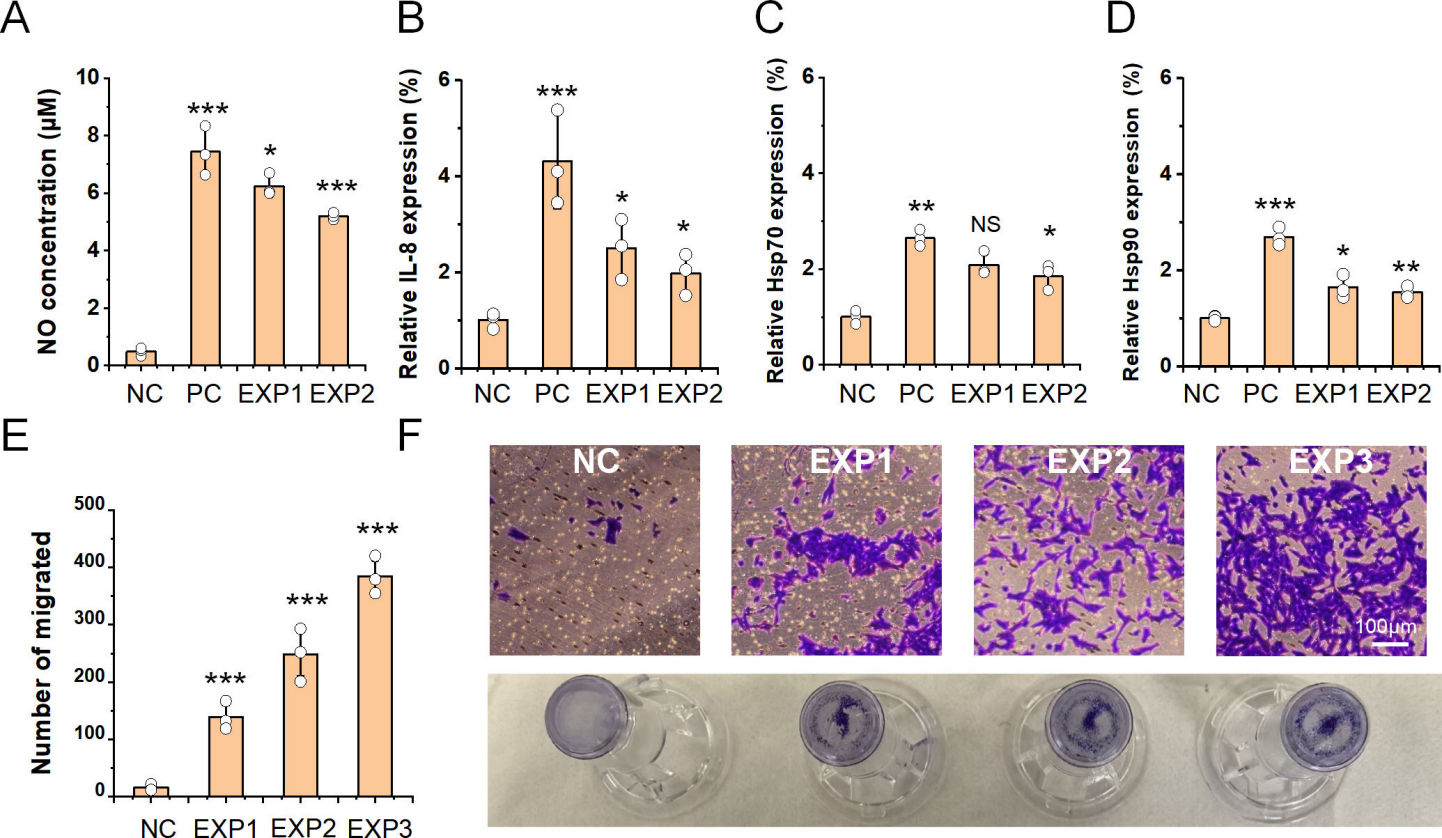
Evaluation of the biological activities of Mcofp-3. (**A**) *In vitro* soothing efficacy assessed via NO production in LPS-induced RAW264.7. (**B**) Anti-inflammatory activity evaluated by measuring the relative mRNA expression of the pro-inflammatory cytokine IL-8 in LPS-induced RAW264.7 mouse macrophages. (**C and D**) Regulation of the heat stress response pathway. Adherent cells were exposed to acute thermal stress to activate Heat Shock Factor 1 (HSF1). The relative mRNA expression levels of the molecular chaperone genes *Hsp70* (**C**) and *Hsp90* (**D**) were quantified to assess the modulatory effect of Mcofp-3. (**E and F**) Effect of Mcofp-3 on the migration of HaCaT keratinocytes using Transwell assays. (**E**) Quantitative analysis of the number of migrated cells. (**F**) Representative microscopy images of HaCaT cells that migrated through an 8-μm pore polycarbonate membrane and attached to the lower surface after 24 h of induction, followed by crystal violet staining. NC, negative control (vehicle); PC, positive control; EXP 1-3, Mcofp-3 at final concentrations of 0.01, 0.02, and 0.05 g L^−1^, respectively (derived from 1% to 5% vol/vol addition of a 1 g L^−1^ Mcofp-3 solution). Statistical significance (*P* values) was determined by two-sided *t*-tests. In panels **A–D**, PC was compared with NC, and EXP1/2 with PC; in panel **E**, EXP1-3 were compared with NC. *P < 0.05, **P < 0.01, *P < 0.001; NS, not significant (*P* ≥ 0.05).

To evaluate the bioactivity of recombinant Mcofp-3, a series of *in vitro* assays was conducted, revealing its significant anti-inflammatory and tissue-repairing properties. Specifically, Mcofp-3 markedly suppressed nitric oxide (NO) overproduction and effectively downregulated the mRNA expression of the pro-inflammatory cytokine IL-8 in LPS-stimulated RAW 264.7 macrophages (Fig. 7A and B), suggesting that it exerts anti-inflammatory effects primarily by inhibiting the NF-κB signaling pathway. In terms of cytoprotection, heat stress response assays demonstrated that Mcofp-3 significantly upregulated the mRNA expression of heat shock protein genes (*Hsp70* and *Hsp90*), confirming its capacity to alleviate thermal injury and promote cellular repair (Fig. 7C and D). Furthermore, scratch wound healing assays indicated that Mcofp-3 effectively accelerated wound closure and skin barrier reconstruction by promoting the migration of both HaCaT keratinocytes and fibroblasts (Fig. 7E and F). Collectively, these findings, supported by statistical analysis (two-sided *t*-test), demonstrate that Mcofp-3 modulates inflammatory responses, cellular stress, and cell migration through a multi-target mechanism, highlighting its immense potential for applications in skin repair, the development of wound dressings, and regenerative medicine.

## DISCUSSION

Mfps are promising for biomedicine due to their strong wet adhesion and biocompatibility, but their recombinant production is hampered by high hydrophobicity, surface stickiness, and cationic residues that cause toxicity in *E. coli* (5, 14, 24). For example, Mcofp-3 expression triggers immediate growth arrest and rapid loss of producing cells (Fig. 1A, 2A and B). This phenomenon stems from the cytotoxicity of Mcofp-3, particularly in soluble or intermediate forms, disrupt host metabolism, triggering stress responses that forcibly terminate expression (39). This mechanism logically explains the observed negative correlation between growth and expression: during the initial induction phase, cells sustain high-level protein expression at the cost of growth arrest, whereas in the late fermentation stage, cells resume growth by halting protein synthesis (Fig. 1D and 2A and B). Current strategies to improve toxic protein expression include induction optimization, fusion tags, sequence engineering, and intracellular compartmentalization—each with notable limitations (40–43). Process optimization only mildly alleviates toxicity, while fusion tags or mutations often compromise the native conformation and function of the target protein (44). In contrast, intracellular compartmentalization, which sequesters toxic proteins into membraneless compartments, offers an effective strategy-physically isolates them from core metabolic pathways, reduces cytosolic free protein concentration, and significantly mitigates cellular damage, representing a key breakthrough for expressing highly toxic proteins (45–49).

HA’s interaction with Mcofp-3 is highly dependent on its molecular weight: only high-molecular-weight HA forms insoluble aggregates *in vitro*, while low-molecular-weight HA yields soluble complexes (Fig. 3A and B). This is because large HA molecules have multiple negative charges and flexible chains that bind strongly to cationic residues (e.g., lysine, arginine) in Mcofp-3, driving network formation and liquid–liquid phase separation. Critically, when the HA biosynthetic pathway was engineered into *E. coli*, intracellularly synthesized HA spontaneously formed dense condensates in the crowded cytoplasm (33, 50) (Fig. 3C). Moreover, the intracellular environment shields HA from extracellular shear forces and extends glycosyl chain elongation time, favoring the accumulation of HMW HA, providing an ideal foundation for our “condensate-based sequestration” strategy (36).

The Mcofp-3/HA co-expression strain grew well after IPTG induction. At late fermentation, Mcofp-3-GFP fluorescence was mainly in granular condensates (Fig. 4A), confirming reduced free protein and lower toxicity. Yields reached 460 mg L^−1^ in shake flasks and scaled smoothly to 1.6 g L^−1^ in 50 L fed-batch fermentation (Fig. 4C and 5B), showing strong scalability. Expression of many proteins, like antimicrobial peptides, cell-penetrating peptides, and amyloids, is limited in prokaryotes by strong charge, hydrophobicity, or membrane disruption (40). By designing intracellular polymers that match their properties (e.g., anionic polysaccharides, cationic polypeptides, or hydrophilic hydrogels), the approach presented in this study enables green, scalable production of diverse high-value toxic proteins. Furthermore, compared to the traditional extraction of Mfps from mussel feet—which is limited by seasonal availability, batch-to-batch variability, and potential marine contaminants—our recombinant platform offers a sustainable, consistent, and animal-free alternative (14).

Additionally, we developed an immobilized tyrosinase, TyrVs–CipA, using the self-assembling tag CipA. The enzyme forms insoluble intracellular aggregates that can be efficiently recovered by simple centrifugation—eliminating complex purification steps—and retains >70% of its initial activity after six consecutive catalytic cycles. This provides an efficient, green, and scalable platform for DOPA modification of Mcofp-3 (51). The resulting DOPA-functionalized Mcofp-3 exhibited potent anti-inflammatory activity and significantly enhanced keratinocyte migration and proliferation, validating its therapeutic potential in skin barrier repair and tissue regeneration (Fig. 7). While the current DOPA modification degree of ~7% validates the system’s functionality, it remains below the levels typically found in native Mfps. To bridge this gap, future efforts will focus on: engineering TyrVs to enhance catalytic efficiency and substrate affinity; optimizing the molar ratio of immobilized enzyme to substrate; and improving the solubility of mussel proteins to facilitate further modification.

In summary, this study elucidates the growth-inhibitory effect of Mcofp-3 in *E. coli* and introduces a “condensate-based sequestration” strategy that leverages polyelectrolyte-driven interactions between HA and Mcofp-3 to achieve targeted capture and spatial sequestration of the protein intracellularly. This approach not only overcomes a major bottleneck in mussel protein biomanufacturing but also establishes a scalable, sustainable, and high-fidelity production paradigm for challenging functional proteins—laying a solid foundation for the industrial application of Mfps.

## MATERIALS AND METHODS

### Strains, plasmids, and reagents

All bacterial strains and plasmids used in this study are listed in Tables S1 and S2, respectively. *E. coli* Top10 was used for routine plasmid construction and amplification, while *E. coli* BL21(DE3) served as the production host for recombinant Mcofp-3, with expression driven by the pET-32a (+) vector.

All primers (Table S3) and gene fragments (*mcofp-3*, *pmHAS*, *gfp*, *CipA*, and *TyrVs*) were synthesized by GENEWIZ (Suzhou, China). Endogenous genes involved in UDP-sugar biosynthesis*—pgm*, *pgi*, *galU*, *ugd*, *glmM*, *glmS*, and *glmU*—were PCR-amplified from genomic DNA of *C. glutamicum* ATCC 13032.

Plasmids were assembled using T5 exonuclease-mediated DNA assembly. All chemicals and molecular biology reagents were sourced from Sangon Biotech (Shanghai, China), Sinopharm Chemical Reagent Co., Ltd., or Takara Bio (Dalian, China), including PrimeSTAR and Ex Taq DNA polymerases and restriction enzymes.

### Strain cultivation and fermentation

*E. coli* strains were routinely grown in Terrific Broth (TB) supplemented with 50 μg mL^−1^ kanamycin or ampicillin. Target protein expression was induced with 0.25 mM IPTG. For shake-flask cultures, a single colony was inoculated into 5 mL TB and incubated overnight at 37°C and 220 rpm. Then, 500 μL of this pre-culture was transferred to 50 mL TB and grown under the same conditions for 8 h to generate a seed culture. In fed-batch fermentation, a 250 mL seed culture was inoculated into 2.5 L TB in a 5-L bioreactor. The culture was maintained at 30°C, with pH controlled at 7.0 ± 0.1 via automatic feeding of 25% (vol/vol) ammonia. Dissolved oxygen (DO) was kept at ~30% by adjusting agitation and aeration, and glycerol was fed as required. IPTG was added at an appropriate cell density to initiate induction.

Tyrosinase-producing recombinant strains were screened by streaking on antibiotic-containing LB indicator plates supplemented with 10 g L^−1^ L-tyrosine, 0.2 mM CuSO₄, and 1 mM IPTG, followed by incubation at 37°C. All shake-flask and bioreactor experiments were performed in three independent biological replicates (*n* = 3), with one analysis per condition per replicate.

### Cell growth and metabolite analysis

Cell density was monitored by measuring the optical density at 600 nm (OD_600_) using a UV-Vis spectrophotometer (Shimadzu, Shanghai, China). Residual glucose concentration was quantified with a glucose analyzer (Thermo Fisher Scientific, Shenzhen, China). Purification and quantification of Mfps: fermentation broth from recombinant *E. coli* was centrifuged at 6,000 × *g* for 8 min to harvest cells, which were resuspended in ultrapure water and lysed by high-pressure homogenization or sonication. The lysate was centrifuged at 12,000 × *g* for 20 min; the supernatant was discarded, and the pellet (inclusion bodies) was resuspended in 25% (vol/vol) ice-cold acetic acid and incubated overnight at 4°C to solubilize Mfps. After a second centrifugation (12,000 × *g*, 20 min), the supernatant was dialyzed to remove salts and small-molecule impurities, then lyophilized to yield crude Mfps. Protein concentration was determined by the Bradford assay. To account for host-derived background, *E. coli* carrying an empty vector was processed in parallel under identical conditions as a negative control. Final Mfps titer was calculated as Mfps yield = (total protein from recombinant strain) – (total protein from empty-vector control).

### Phase-contrast microscopy and TEM analysis

Phase-contrast and fluorescence imaging were performed using an Eclipse Ni-E microscope (Nikon, Tokyo, Japan) equipped with a GFP filter set (excitation: 465–495 nm; dichroic mirror: 505 nm; emission: long-pass >512 nm). Transmission electron microscopy (TEM) samples were prepared following standard protocols: double fixation with glutaraldehyde and osmium tetroxide, graded ethanol dehydration, embedding in EPON 812 resin, ultrathin sectioning, and lead citrate staining (33). Samples were examined using a Hitachi H7650 TEM (80 kV, Tokyo, Japan). Acquired images were analyzed using ImageJ software (Bethesda, MD, USA).

### HA quantification and mass spectrometry analysis

HA was purified as follows: intracellular HA was recovered from lysed cells (by sonication or high-pressure homogenization) through repeated ethanol precipitation. The HA concentration in purified samples was quantified using the carbazole-sulfuric acid assay, with D-glucuronic acid as the external standard. *E. coli* carrying an empty vector was processed in parallel as a negative control to correct for background signals.

For structural characterization, recombinantly expressed leech-derived hyaluronidase was used to fully depolymerize HA (52, 53). The resulting digest was mixed with nine volumes of methanol, filtered, and analyzed by LC-IT-TOF-MS (Shimadzu, Kyoto, Japan) under chromatographic and mass spectrometric conditions adapted from published protocols.

### *In vitro* interaction between Mcofp-3 and multivalent polyanionic compounds

Reagent preparation: weigh an appropriate amount of polyanionic compounds, including hyaluronic acid, chondroitin sulfate C, chondroitin sulfate A, heparin, sodium alginate, dextran, and polyphosphoric acid, dissolve them in water, and fix the volume to prepare a stock solution of 5 g L^−1^; meanwhile, prepare a 1 g L^−1^ Mcofp-3 protein solution. Take a centrifuge tube, add 2 volumes of Mcofp-3 protein solution and 1 volume of each polyanionic stock solution, invert gently to mix evenly (avoid vigorous shaking), incubate statically at room temperature, observe and record whether precipitation occurs in the system, the morphology of the precipitation, and the degree of turbidity, with water as the negative control.

### IL-8 gene expression assay

RAW 264.7 mouse macrophages were cultured in DMEM with 10% FBS and 1% penicillin–streptomycin at 37°C, 5% CO_2_. Three groups were tested: negative control (NC, medium only), LPS model (PC, 5 μg mL^−1^ LPS for 18 h), and experimental group (EXP, sample + LPS). After 18–24 h pre-culture in 6-well plates, cells were primed with LPS for 6 h, then the medium was replaced—EXP with sample-containing medium, PC and NC with fresh plain medium—and incubated another 24 h. IL-8 mRNA levels were measured by RT-qPCR (GAPDH as reference; primer sequences provided GAPDH-F/R, 5′-CTCCCACTCTTCCACCTTCG/TTGCTGTAGCCGTATTCATT-3′; IL-8-F/R, 5′-GAGAGTGATTGAGAGTGGACCAC/CACAACCCTCTGCACCCAGTTT-3′) and expressed relative to the PC group (set as 100%). Data are shown as mean ± SD (*n* = 3). Experiments were valid only if PC showed significantly higher IL-8 than NC (*P* < 0.05, *t*-test). Statistical analysis was performed using SPSS.

### Total NO assay

Third-passage mouse RAW264.7 cells (Procell) were cultured in DMEM supplemented with 10% FBS and 1% penicillin–streptomycin at 37°C, 5% CO₂, and saturated humidity. Three groups were tested: NC (medium only), PC (5 μg mL^−1^ LPS for 18 h), and EXP (sample + LPS for 18 h).

Cells were seeded in 6-well plates and pre-cultured for 18–24 h. PC and EXP groups were then treated with LPS for 18 h. The medium was subsequently replaced—EXP with fresh medium containing the test sample, and PC/NC with plain medium—and incubation continued for 24 ± 2 h. Culture supernatants were collected, centrifuged, and nitric oxide (NO) levels were measured using a commercial NO assay kit per the manufacturer’s instructions. Each group included four biological replicates, and results are presented as mean ± SD.

### Transwell cell migration assay

Log-phase HaCaT cells (Haixing Bio, Suzhou) were resuspended in serum-free H-DMEM at 2.5 × 10⁵ cells mL^−1^. In a 24-well plate, 300 μL complete medium (10% FBS) was added per well, and an 8-μm pore-size Transwell insert was placed into each well (54). The upper chamber was seeded with 200 μL cell suspension (5 × 10⁴ cells/well), followed by 1 h serum starvation at 37°C. The medium was then replaced with serum-free medium containing the specified treatment: NC (medium only) and EXP (sample + medium), and cells were incubated for 24 h.

Transwell inserts were removed, rinsed once with PBS, and non-migrated cells on the upper surface were gently wiped off with a cotton swab. Cells were fixed with 4% paraformaldehyde for 30 min, stained with 0.1% crystal violet for 20 min, washed three times with PBS, and residual dye on the upper side was carefully removed. Migrated cells on the lower surface were imaged under an inverted microscope (Zeiss, Germany) and counted in multiple random fields per insert.

### Heat shock stress assay

Adherent cells at log phase (~1 × 10⁶ cells/well) were washed and incubated in pre-warmed, antibiotic-free medium. Dishes were sealed with Parafilm and heat-shocked at 50°C for 30 min with gentle shaking every 5 min. After heat shock, the medium was replaced with fresh, pre-warmed complete medium containing the specific treatments for each group: NC (medium only), PC (heat shock at 50°C for 30 min), and EXP (heat shock at 50°C for 30 min plus sample treatment); cells were then incubated at 37°C, 5% CO_2_ for 6 h. Total RNA was then extracted, reverse-transcribed, and *hsp70* and *hsp90* expression was analyzed by qPCR (55).

### DOPA modification, qualitative detection, and quantification

DOPA modification reaction: in a 1 mL reaction mixture, 1 mg mL^−1^ Mfp, 0.1 mM CuSO_4_, and immobilized TyrVs–CipA enzyme (pellet from lysed cells, adjusted to OD_600_ = 10.0) were combined. The reaction was carried out at 37°C with shaking at 220 rpm for 30 min. After centrifugation, the supernatant containing the DOPA-modified product was collected, and the enzyme pellet was recovered for reuse. Qualitative DOPA detection (NBT assay): 2 μL of sample was spotted onto a 0.2 μm nitrocellulose membrane and sonicated in 300 mL water for 10 min. The membrane was then incubated in NBT staining solution (9 mg NBT dissolved in 15 mL glycine–KOH buffer, pH 10) in the dark for 45 min. After two washes with sodium borate solution (2.5 g in 40 mL), the membrane was soaked overnight in the same borate solution in the dark. The next day, it was rinsed three times with water and examined for blue-purple spots indicative of DOPA. Quantitative DOPA determination (colorimetric assay): 1 mL of sample or standard solution was mixed sequentially with 0.5 mL of 0.516 mM HCl and 1.5 mL of nitrite reagent (containing 10% sodium molybdate and 10% sodium nitrite). Within 5 min, 2 mL of 1 mM NaOH was added, and the mixture was incubated at room temperature. Absorbance was measured at 500 nm against a blank. Turbid samples were first centrifuged at 3,000 rpm for 10 min, and the supernatant was used; if absorbance exceeded the linear range, samples were diluted accordingly. DOPA concentration was calculated based on a standard curve.

## Data Availability

The data supporting this article have been included as supplemental material. Any other data will be made available on request.

## SUPPLEMENTAL MATERIAL

Supplemental material aem.00786-26-s0001.pdf Fig. S1 to S11; Tables S1 to S3.

ASM does not own the copyrights to Supplemental Material that may be linked to, or accessed through, an article. The authors have granted ASM a non-exclusive, world-wide license to publish the Supplemental Material files. Please contact the corresponding author directly for reuse.
