# Supplementary material for: Condensate-based sequestration enables high-efficiency synthesis of mussel foot protein Mcofp-3
Source: Appl Environ Microbiol. 2026 Jun 4;92(7):e00786-26. doi: 10.1128/aem.00786-26 (PMC13390468; doi:10.1128/aem.00786-26)
Supplement: Supplemental material — Fig. S1 to S11; Tables S1 to S3. [file aem.00786-26-s0001.pdf]

Supplementary Materials for

Condensate-based sequestration enables high-efficiency synthesis of mussel foot protein Mcofp-3

Litao Hu [1,4], Liyan Chen [1,4], Yan Wang [1], Siting Yu [2,4], Sen Xiao [2,3,4], Wuxia Chen[4], Jianhua Cheng [1,4*], Zhen Kang [2,3,4*]

[1]College of Environment and Energy, South China University of Technology, Guangzhou, 510006, China

[2]The Key Laboratory of Carbohydrate Chemistry and Biotechnology, Ministry of Education, School of Biotechnology, Jiangnan University, Wuxi, 214122, China

[3]The Science Center for Future Foods, Jiangnan University, Wuxi, 214122, China

[4]Institute of Future Food Technology, JITRI, Yixing 214200, China

*Correspondence should be addressed to Z.K (zkang@jiangnan.edu.cn) and J.C (jhcheng@scut.edu.cn)

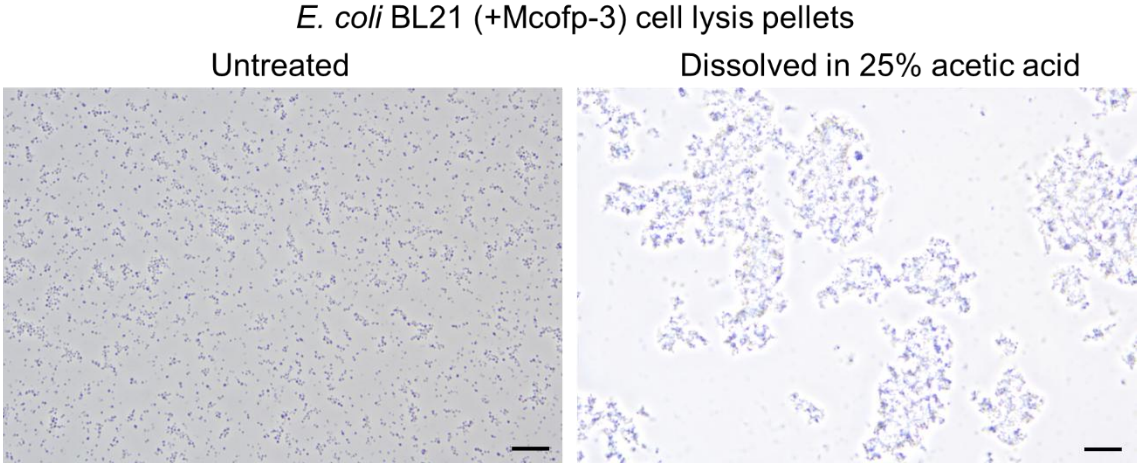

Figure S1. Microscopic images of *E. coli* BL21 (+Mcofp-3) cell lysis pellets: (Left) untreated pellet; (Right) pellet dissolved in 25% acetic acid for 5 min, Scale bars: 10 μm.

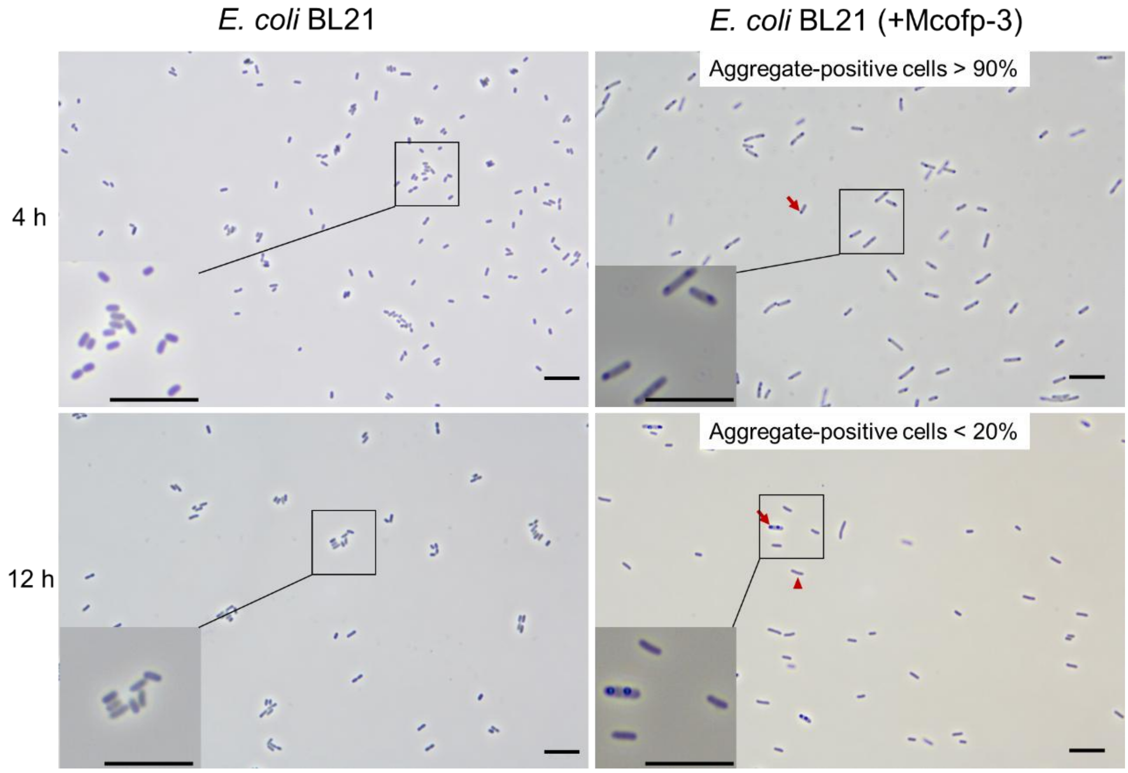

Figure S2. Phase-contrast microscopy images of recombinant Mcofp-3 strains at different growth stages, with *E. coli* BL21 harboring an empty vector as a control. Arrows indicate intracellular mussel protein aggregates, while arrowheads denote cells lacking Mcofp-3, Scale bars: 10 μm.

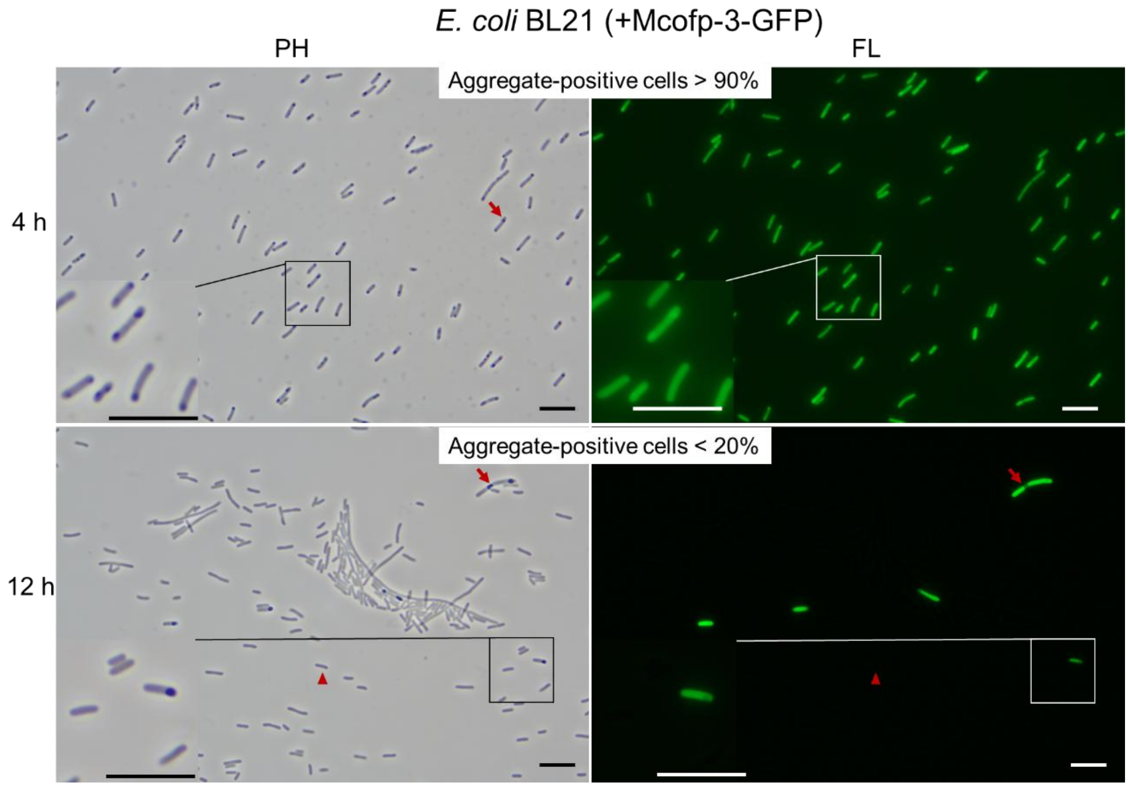

Figure S3. Phase-contrast and fluorescence microscopy images of the Mcofp-3-GFP strain, Scale bars: 10 μm.

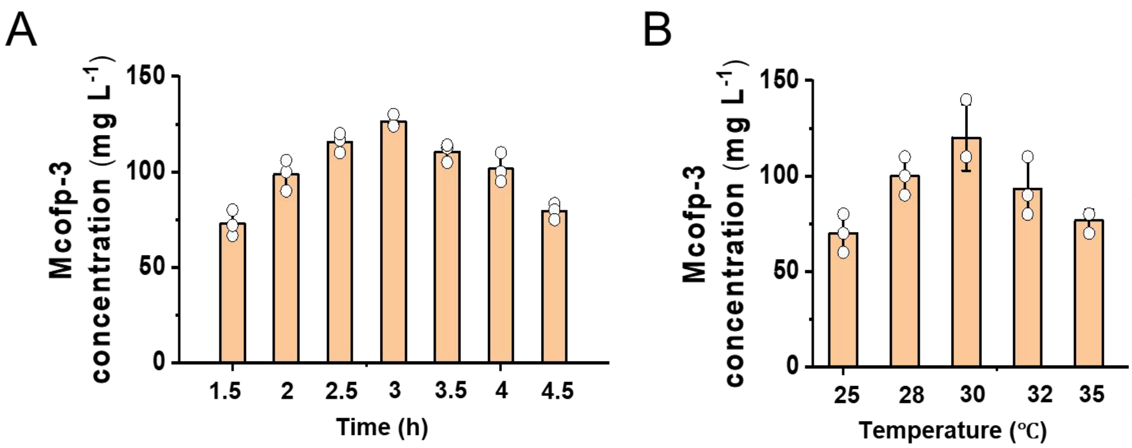

Figure S4. Enhancement of Mcofp-3 yield by optimizing IPTG induction time and fermentation temperature. (A) Mcofp-3 yield at different IPTG induction times (1.5, 2, 2.5, 3, 3.5, 4, 4.5 h) with a fixed IPTG concentration of 1 mM. (B) Mcofp-3 yield at different fermentation temperatures (25, 28, 30, 32, 35 °C, etc.) with a fixed IPTG concentration of 1 mM and an induction time of 1 h.

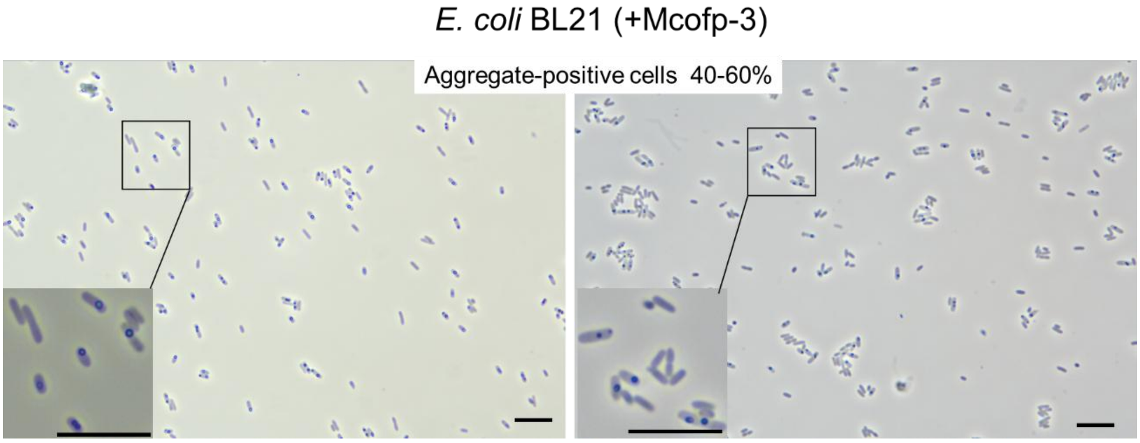

Figure S5. Microscopic images of *E. coli* BL21 (+Mcofp-3) cells after 12 h of fermentation under optimized induction conditions (0.1 mM IPTG, 3 h induction, 30 °C), Scale bars: 10 μm.

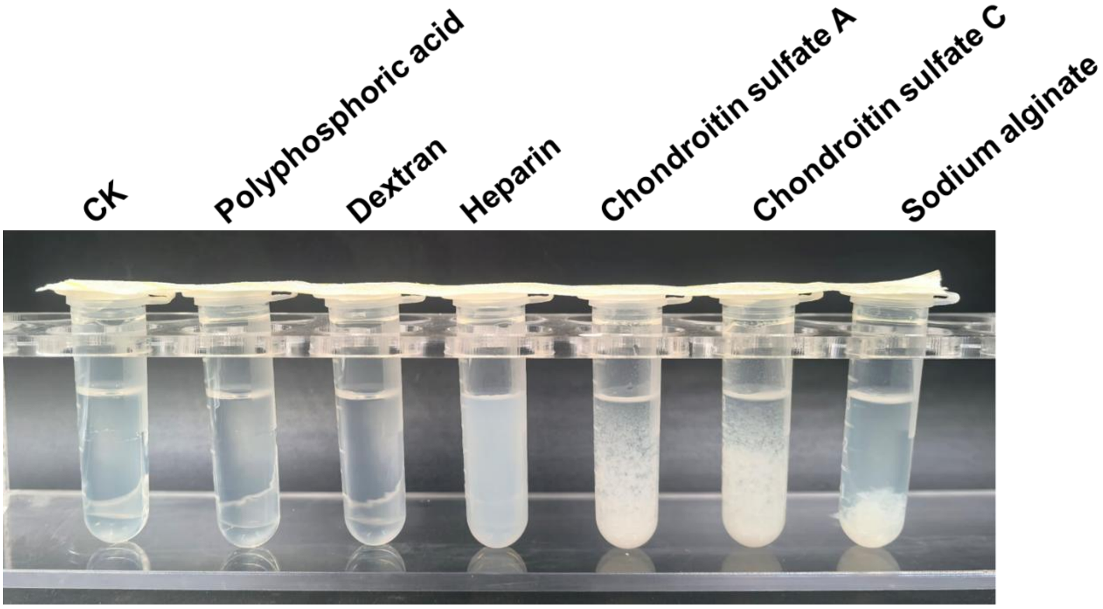

Figure S6. Visual confirmation of specific interactions. Mixtures containing 5 g L^{-1} multivalent polyanionic polymers (polyphosphoric acid, dextran, heparin, chondroitin sulfate C, chondroitin sulfate A, and sodium alginate) and 1 g L^{-1} Mcofp-3 exhibit distinct phase separation behaviors.

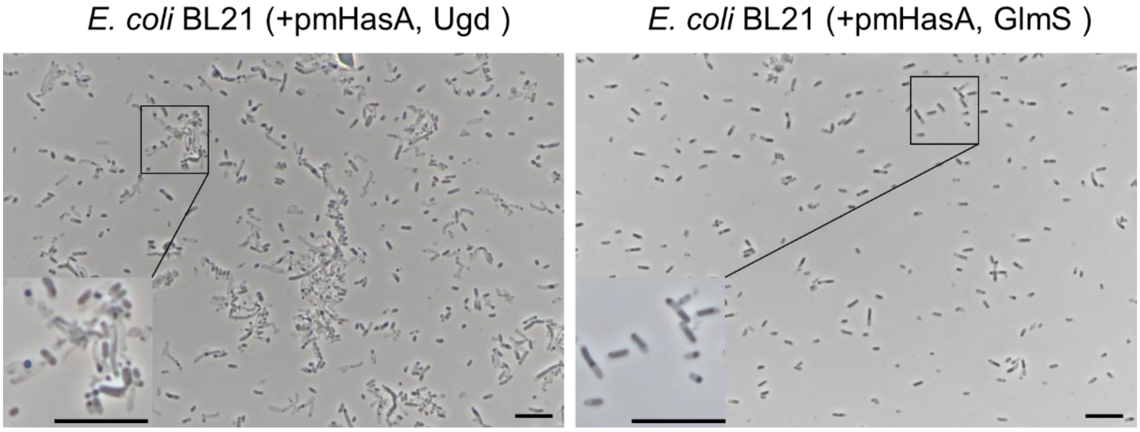

Figure S7. Phase-contrast micrographs of *E. coli* BL21 strains expressing *pmHasA* with *glmS* or *ugd* after 12 h of fermentation, Scale bars: 10 μm.

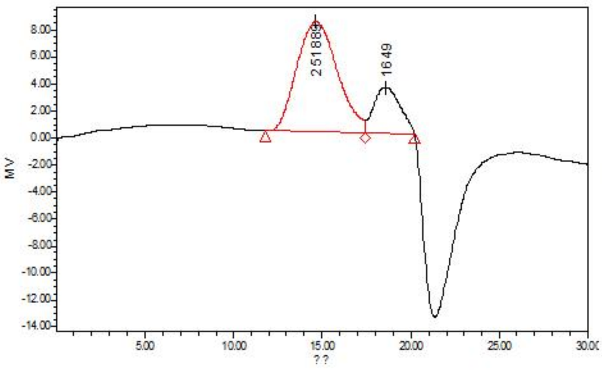

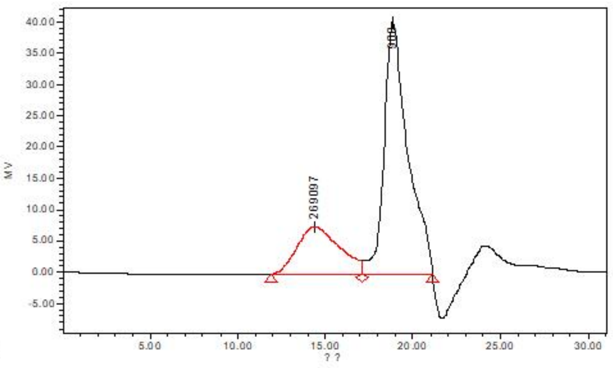

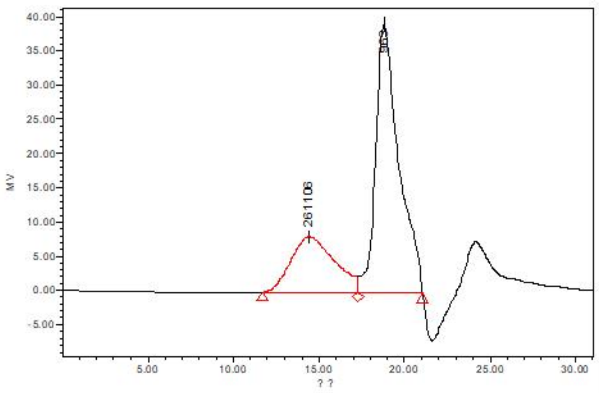

Sample	Mn	Mw	MP
1	85626	430413	251889
2	71898	399286	269097
3	60186	416053	261106
Average	78762	414849.5	260493
Stdev	12733	15579	8611

Figure S8. Molecular weight distribution of intracellular HA from *E. coli* BL21 (*pmHasA* + *glmS*) after 12 h fermentation.

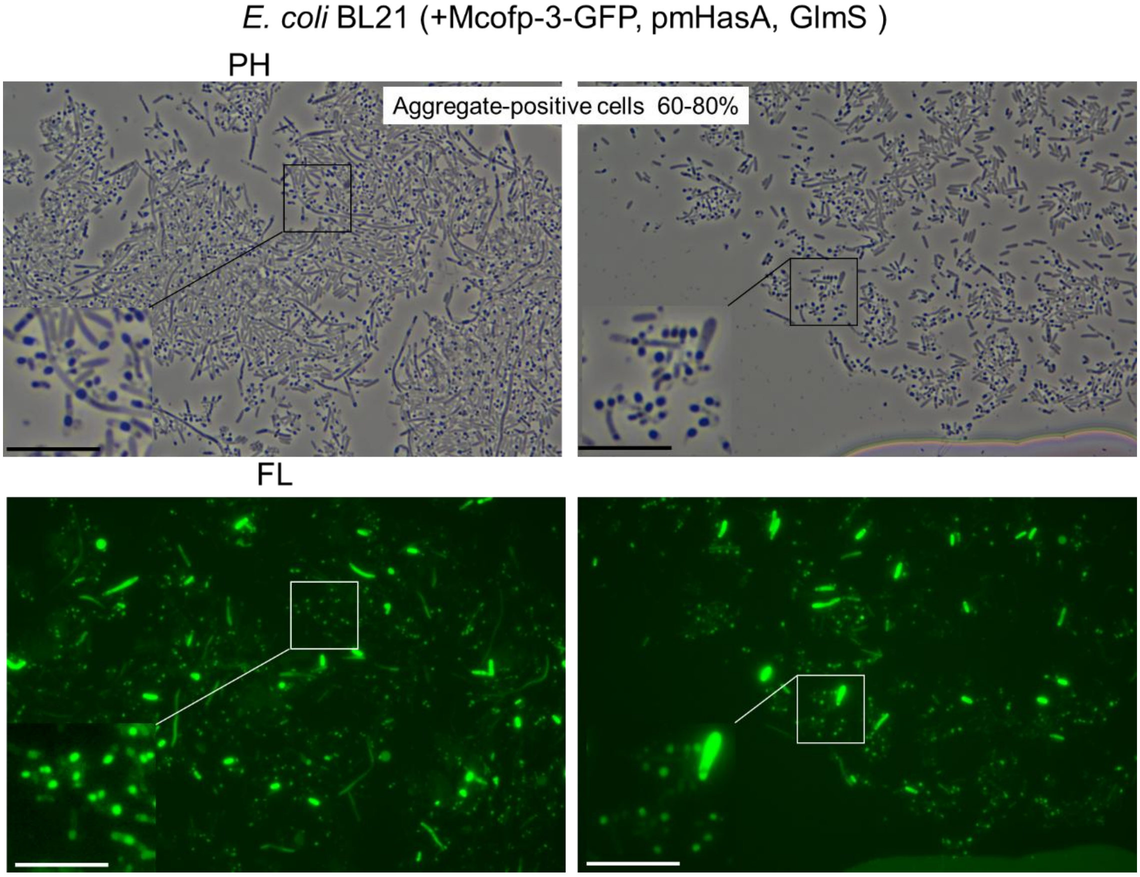

Figure S9. Phase-contrast and fluorescence microscopy images of the engineered strain co-expressing *mcofp-3-gfp*, *pmHasA*, and *glmS*, Scale bars: 10 μm.

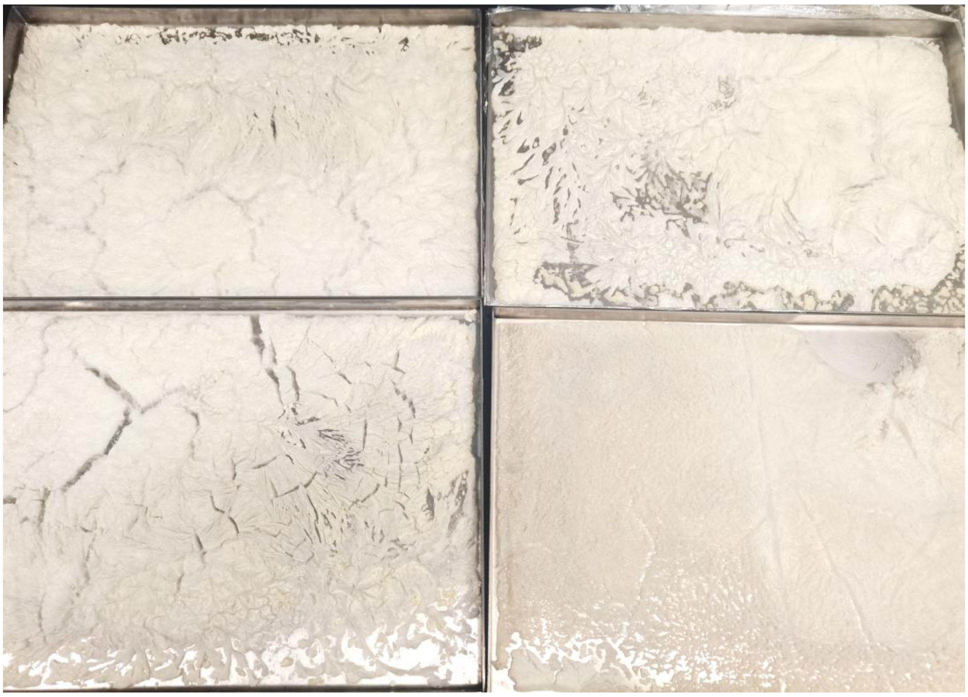

Figure S10. Purified and freeze-dried Mcofp-3 samples obtained from cell lysates harvested from a 50 L fed-batch fermentation.

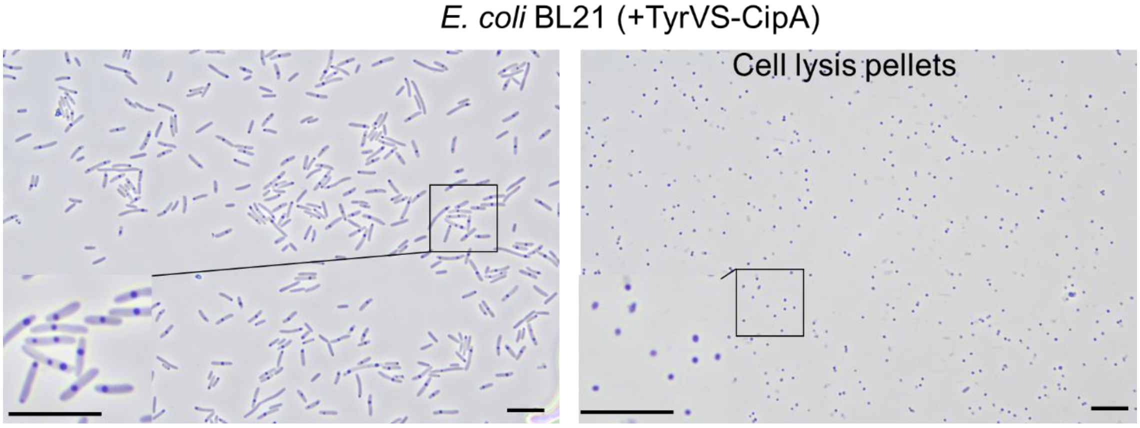

Figure S11. Microscopic images of *E. coli* BL21 (+TyrVS-CipA) (Left) and cell lysis pellets (Right), Scale bars: 10 μm.

Table S1. Plasmids used in this study

Plasmids	Description	Source
pET32a	*E. coli* expression vector, T7 promoter, kanamycin resistance, ColE1 replicon	Lab stock
pET32a-mcofp-3	pET32a-plasmid expression of the mussel foot protein Mcofp-3 from *Mytilus coruscus*	This study
pET32a-mcofp-3-gfp	pET32a-plasmid expression of the mussel foot protein Mcofp-3-linked to green fluorescent protein (GFP)	This study
pXMJ19-pmhasA	pXMJ19 plasmid expressing hyaluronic acid synthase gene of *Pasteurella multocida, pmhasA*	This study
pXMJ19-pmhasA-Ugd	pXMJ19 plasmid expressing hyaluronic acid synthase gene of *P. multocida*, *pmhasA* and the UDP-glucose dehydrogenase gene (ugd) from *Corynebacterium glutamicum*	This study
pXMJ19-pmhasA-pgm	pXMJ19 plasmid expressing hyaluronic acid synthase gene of *P. multocida*, *pmhasA* and the phosphoglucomutase (pgm) gene from *C. glutamicum*	This study
pXMJ19-pmhasA-galU	pXMJ19 plasmid expressing hyaluronic acid synthase gene of *P. multocida*, *pmhasA* and the glucose-1-phosphate uridylyltransferase gene (*galU*) from *C. glutamicum*	This study
pXMJ19-pmhasA-pgi	pXMJ19 plasmid expressing hyaluronic acid synthase gene of *P. multocida*, *pmhasA* and the glucose-6-phosphate isomerase gene (*pgi*) from *C. glutamicum*	This study
pXMJ19-pmhasA-glmS	pXMJ19 plasmid expressing hyaluronic acid synthase gene of *P. multocida*, *pmhasA* and the L-glutamine-D-fructose-6-phosphate aminotransferase gene (*glmS*) from *C. glutamicum*	This study
pXMJ19-pmhasA-glmM	pXMJ19 plasmid expressing hyaluronic acid synthase gene of *P. multocida*, *pmhasA* and the phosphoglucosamine mutase gene (*glmM*) from *C. glutamicum*	This study
pXMJ19-pmhasA-glmU	pXMJ19 plasmid expressing hyaluronic acid synthase gene of *P. multocida*, *pmhasA* and the UDP-N-acetylglucosamine pyrophosphorylase/Glucosamine-1-phosphate Nacetyltransferase gene (*glmU*) from *C. glutamicum*	This study
pET28a-tyrVs	pET32a plasmid expressing of *V. spinosum* Tyrosinase	This study
pET28a-tyrVs-CipA	pET32a plasmid expressing Tyrosinase TyrVs gene fused to the C-terminus of the *cipA* gene	This study

Table S2. Strains used in this study

Strains	Description	Source
E. coli TOP10	*E. coli TOP10 (Genotype: F⁻ araD139 Δ(ara-leu)7697 ΔlacX74 φ80lacZΔM15 recA1 endA1 rpsL galU galK Δ(mrr-hsdRMS-mcrBC) mcrA nupG; Thermo Fisher Scientific)*	Lab stock
E. coli BL21	*E. coli* BL21(DE3)	Lab stock
EC-Mcofp-3	*E. coli* BL21 derivate, harboring pET32a-mcofp-3	This study
EC-Mcofp-3-GFP	*E. coli* BL21 derivate, harboring pET32a-mcofp-3-gfp	This study
EC01	*E. coli* BL21 derivate, harboring pXMJ19-pmhasA	This study
EC02	*E. coli* BL21 derivate, harboring pXMJ19-pmhasA-Ugd	This study
EC03	*E. coli* BL21 derivate, harboring pXMJ19-pmhasA-pgm	This study
EC04	*E. coli* BL21 derivate, harboring pXMJ19-pmhasA-galu	This study
EC05	*E. coli* BL21 derivate, harboring pXMJ19-pmhasA-pgi	This study
EC06	*E. coli* BL21 derivate, harboring pXMJ19-pmhasA-glms	This study
EC07	*E. coli* BL21 derivate, harboring pXMJ19-pmhasA-glmm	This study
EC08	*E. coli* BL21 derivate, harboring pXMJ19-pmhasA-glmu	This study
EC09	*E. coli* BL21 derivate, harboring pET32a-mcofp-3 and pXMJ19-pmhasA-glms	This study
EC10	*E. coli* BL21 derivate, harboring pET32a-mcofp-3-gfp and pXMJ19-pmhasA-glms	This study
EC11	*E. coli* BL21 derivate, harboring pET32a-tyrVs	This study
EC12	*E. coli* BL21 derivate, harboring pET32a-tyrVs-CipA	This study

Table S3. Primers used in this study

Name	Sequence (5' to 3')
32a-R	GGATCCGTGGTGGTGGTGGTGGTGCATATGTATATCTCCTTCTTAAAGT TAAACAAAATTATTTCTAGAGGG
32a-F	GGATCCGAATTCGAGCTC
Mcofp-R	GTCGACGGAGCTCGAATTCGGATCCTTAGTACAGGTAACCTTTGTTAC C
Mcofp-F	CACCACCACCACCACGGATCCGATGCGGGTTATGGTTACTATCCGG
Gfp-F	AAGGTTACCTGTACTAACTCGAGAAAAGGAGGAAAAAAAATGGGTAAG GGAGAAGAACTT
Gfp-R	CGACGGAGCTCGAATTCGGATCCTTATTTGTACAGTTCATCCATGCCAT GTGTAAT
Mcofp-Gfp-F	GGATCCGAATTCGAGCTC
Mcofp-Gfp-R	TTTTCTCGAGTTAGTACAGGTAACCTTTGTTACCGTAGTAAGAACCACC CCACGGGCCAGAGTTCCAACCTT
Samn-R	GGTACCTTATAGAGTTATACTATTAATAATG
Samn-F	GAGCTCGAATTCAGCTTGGC
Galu-F	ATAGTATAACTCTATAAGGTACCAAGGAGGCATTTACATATGAGTTTGCC TATCGATGAG
Galu-R	GCCAAGCTGAATTCGAGCTCCTATTTTACTTGAGAATCGTCTGC
Ugd-F	ATAGTATAACTCTATAAGGTACCAAGGAGGCATTTACATATGCGGATGAC AGTGATTGGTAC
Ugd-R	GCCAAGCTGAATTCGAGCTCCTAAAGGTTGCGGCCGAGCGC
GlmM-F	ATAGTATAACTCTATAAGGTACCAAGGAGGCATTTACATATGACTCGACT ATTTGGAACTGATGG
GlmM-R	GCCAAGCTGAATTCGAGCTCTTAGACTTCTGCAACCACTGCA
GlmS-F	ATAGTATAACTCTATAAGGTACCAAGGAGGCATTTACATATGCGCATGTG TGGAATTGTTGG
GlmS-R	GCCAAGCTGAATTCGAGCTCTTATTCGACGGTGACAGACTTTGC
GlmU-F	ATAGTATAACTCTATAAGGTACCAAGGAGGCATTTACATATGAAATCAGA CTTACAAAAAACGTTGC
GlmU-R	GCCAAGCTGAATTCGAGCTCTTAGCCTTCCTGGTTGTGGACGTTTTG
C-CipA(28a)-F	GAAATCTCTATGTAAGAATTCGAGCTCCGTCGACAAGC
C-CipA(28a)-R	GTGGTATTTCGCCATTATATCTCCTTCTTTCTAGAGGGGAATTGTTATCC
TyrVs-C-F	ATGGCGAAATACCACCGTCTGA
TyrVs-C-R	CATGTCATTAATCATGGATCCTCCTCCTCCGGATCCTCCTCCTCCGGAT CCTC CTCCTCCTTTGTTGTTCATTTCACCCGGACGA
